# White matter degeneration in subjective cognitive decline: a diffusion tensor imaging study

**DOI:** 10.18632/oncotarget.10091

**Published:** 2016-06-15

**Authors:** Xuan-yu Li, Zhen-chao Tang, Yu Sun, Jie Tian, Zhen-yu Liu, Ying Han

**Affiliations:** ^1^ Department of Neurology, XuanWu Hospital of Capital Medical University, Beijing, 100053, China; ^2^ Key Laboratory of Molecular Imaging, Institute of Automation, Chinese Academy of Sciences, Beijing, 100190, China; ^3^ School of Mechanical, Electrical & Information Engineering, Shandong University, Weihai, Shandong Province, 264209, China; ^4^ Center of Alzheimer's Disease, Beijing Institute for Brain Disorders, Beijing, 100053, China

**Keywords:** subjective cognitive decline, diffusion tensor imaging, preclinical Alzheimer's disease, white matter, tract-based spatial statistics

## Abstract

Subjective cognitive decline (SCD) may be an at-risk stage of Alzheimer's disease (AD) occurring prior to amnestic mild cognitive impairment (aMCI). To examine white matter (WM) defects in SCD, diffusion images from 27 SCD (age=65.3±8.0), 35 aMCI (age=69.2±8.6) and 25 AD patients (age=68.3±9.4) and 37 normal controls (NC) (age=65.1±6.8) were compared using Tract-Based Spatial Statistics (TBSS). WM impairments common to the three patient groups were extracted, and fractional anisotropy (FA) values were averaged in each group. As compared to NC subjects, SCD patients displayed widespread WM alterations represented by decreased FA (p<0.05), increased mean diffusivity (MD; p<0.05), and increased radial diffusivity (RD; p<0.05). In addition, localized WM alterations showed increased axial diffusivity (AxD; p<0.05) similar to what was observed in aMCI and AD patients (p<0.05). In the shared WM impairment tracts, SCD patients had FA values between the NC group and the other two patient groups. In the NC and SCD groups, the AVLT-delayed recall score correlated with higher AxD (r=−0.333, p=0.045), MD (r=−0.351, p=0.03) and RD (r=−0.353, p=0.025). In both the aMCI and AD groups the diffusion parameters were highly correlated with cognitive scores. Our study suggests that SCD patients present with widespread WM changes, which may contribute to the early memory decline they experience.

## INTRODUCTION

Subjective cognitive decline (SCD), the self-perception of cognitive decline without objective evidence on standardized neuropsychological tests, is increasingly considered an at-risk stage of Alzheimer's disease (AD), predating Mild Cognitive Impairment (MCI) [1]. A recent meta-analysis suggested that the annual conversion rates from SCD to MCI or AD are approximately 6.6% and 2.3%, respectively [2]. SCD may be the sole symptom for which patients seek medical advice and may be an opportunity for early intervention. Compared with MCI, SCD patients have only mild neurodegeneration and increased likelihood of successful functional compensation. Research on the SCD stage may help us better understand the early pathological mechanisms of AD, since the underlying pathological process of AD begins decades before its diagnosis. AD-related biomarkers in SCD patients would therefore be of great value [3–8].

Previous studies have suggested that cerebrospinal fluid biomarkers, including low amyloidβ-42 (Aβ-42) and high tau levels, are more common in SCD patients compared with normal controls (NC), and that low Aβ-42 may be a good predictor of clinical progression in SCD [9, 10]. SCD patients have AD-like gray matter changes in conventional structural magnetic resonance imaging (MRI) and activity differences on functional MRI [11–17]. However, white matter (WM) changes in SCD are not well studied and remain debated [18–23]. Diffusion tensor imaging (DTI) is a quantitative MRI technique that has been applied to detect alterations in WM [24]. There are several parameters derived from DTI which describe different aspects of diffusion tensors. Fractional anisotropy (FA) and mean diffusivity (MD) characterize the water diffusion in the tissue, and are used to describe the WM structural integrity [25–27]. Also, axial diffusivity (AxD) and radial diffusivity (RD) may provide additional information about axonal degeneration and demyelination, respectively [27–30].

Using DTI measures, Selnes and colleagues observed higher RD and MD in those WM tracts underlying the retrosplenial, posterior cingulate, and middle temporal cortices in the SCD group when compared to the NC group [19]. In their follow-up study, they proposed that DTI predicts medial temporal lobe atrophy and dementia [18]. In contrast to these positive results, two other studies found no difference between SCD and normal aging groups by DTI, though Wang and colleagues suggested that SCD showed an intermediate pattern between NC and MCI groups [22, 23]. There are numerous causes of SCD that are not related to AD, such as normal aging, psychiatric and neurologic disorders, medication and substance abuse. Thus, SCD patient heterogeneity may partly explain discrepancies in results. In 2014, the Subjective Cognitive Decline Initiative (SCD-I) Working Group proposed research criteria for pre-MCI SCD in order to standardize future research on SCD. They list several specific features of SCD that would increase the possibility of preclinical AD, according to current data. These criteria gave us the opportunity to verify previous DTI studies in screened SCD subjects and further explore the relationship between DTI parameters and their cognitive performance.

In the present study, we examined the WM tract diffusivity of normal aging, SCD, aMCI, and AD subjects using a 3.0T MRI scanner, and correlated these findings with neuropsychological test scores. We hypothesized that the SCD patients would show milder WM impairment than aMCI and AD patients and that these WM changes would correlate with cognitive dysfunction.

## RESULTS

### Subject characteristics

Demographics and neuropsychological performance of the four groups were summarized in Table 1. There were no differences between the four groups in age, gender or years of education. As expected, the NC and SCD groups showed similar performance on the Mini-Mental State Examination (MMSE) and Montreal Cognitive Assessment (MoCA), while the aMCI and AD groups had significantly lower MMSE and MoCA scores than the NC and SCD groups. There were differences among all the groups on scores of Auditory Verbal Learning test (AVLT) immediate recall, delayed recall, and recognition. Although the memory performance of the SCD group was intermediate to the NC and other two patient groups, it was still considered within the normal range.

**Table 1:** Demographics and neuropsychological performance of patients and controls

	NC (n=37)	SCD (n=27)	aMCI (n=35)	AD (n=25)	P value
Age, years	65.1±6.8	65.3±8.0	69.2±8.6	68.3±9.4	0.107
Gender (M/F)	14/23	9/18	25/10	8/16	0.527
Education	9.9±4.5	11.4±3.9	8.9±4.3	10.0±4.6	0.157
CDR	0±0	0±0	0.5±0	1±0	-
MMSE	27.5±1.9^c^^d^	27.6±1.6^c^^d^	24.6±3.8^a^^b^^d^	17.6±6.0^a^^b^^c^	<0.001
MoCA	26.2±3.0^c^^d^	26.1±2.8^c^^d^	19.7±4.2^a^^b^^d^	13.9±5.4^a^^b^^c^	<0.001
AVLT-immediate recall	26.9±5.1^b^^c^^d^	23.5±4.4^a^^c^^d^	16.9±4.4^a^^b^^d^	10.5±4.5^a^^b^^c^	<0.001
AVLT-delayed recall	9.6±2.5^b^^c^^d^	7.7±2.3^a^^c^^d^	3.7±2.8^a^^b^^d^	0.68±1.3^a^^b^^c^	<0.001
AVLT-recognition	11.7±2.6^c^^d^	10.7±1.9^c^^d^	7.6±3.9^a^^b^^d^	3.6±3.0^a^^b^^c^	<0.001

Data are presented as the mean ± SD. NC, normal control; SCD, Subjective cognitive decline; aMCI, Amnestic Mild Cognitive Impairment; AD, Alzheimer's disease; CDR: Clinical Dementia Rating Scale; MMSE, Mini-Mental State Examination; MoCA, Montreal Cognitive Assessment; AVLT, Auditory Verbal Learning Test.

a Indicates significant differences compared with the control group;

b Indicates significant differences compared with the SCD group;

c Indicates significant differences compared with the aMCI group;

d Indicates significant differences compared with the AD group (ANOVA, covariates: age, years of education, depression MMSE scores; Chi square test, covariate: gender). The threshold was set at P<0.05.

### WM changes in each patient group

**Figure 1:**
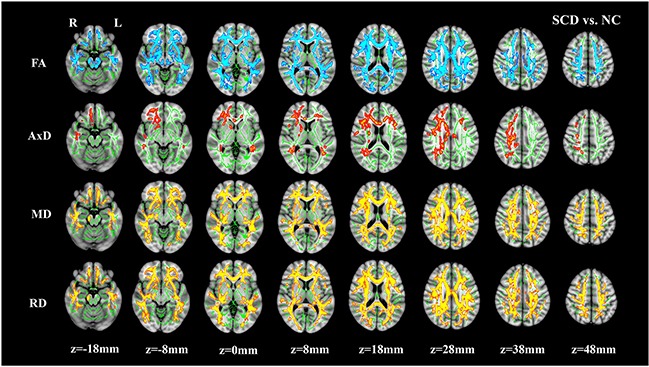
Group differences between normal controls (NC) and subjective cognitive decline (SCD) The brain images showing underlying standard Montreal Neurological Institute (MNI) atlas MNI152 1-mm brain template and white matter skeleton derived from tract-based spatial statistics (TBSS) analysis (shown in green). Blue-Light blue color indicates tracts with decreased fractional anisotropy (FA), Red-Yellow color indicates tracts with increased mean diffusivity (MD) and radial diffusivity (RD) in SCD vs. NC, respectively. For axial diffusivity (AxD), no voxels were significantly different between SCD and NC. The threshold for results was set at P<0.05 (TFCE and FWE corrected, voxels>100).

Widespread WM impairment (represented by DTI parameter alterations) was observed in the SCD, aMCI, and AD groups after Family-wise error (FWE) correction (Figure 1, Supplementary Figures S1&S2). Compared to NC, the SCD group had decreased FA, and increased MD and RD in widespread WM tracts, including the body, genu, and splenium of the corpus callosum, the internal capsule, the external capsule, the corona radiata, the superior longitudinal fasciculus, the superior fronto-occipital fasciculus, the fornix, the uncinate fasciculus, the cingulate gyrus, the posterior thalamic radiation, the tapetum, and the sagittal striatum (Figure 1, Supplementary Table S1). In addition, increased AxD were found in more localized regions including the internal capsule, the corona radiata, the posterior thalamic radiation, the sagittal striatum, the cingulate gyrus, the superior longitudinal fasciculus, and the tapetum in the SCD group. The impaired WM tracts of the aMCI and AD groups represented by decreased FA and increased MD, AxD and RD were roughly the same as the SCD patients (Supplementary Figures S1&S2, Supplementary Tables S2&S3). Additionally, the results surviving P<0.005 (TFCE and FWE corrected, voxels >500) threshold are present in Supplementary Figures S3-S5 and Supplementary Tables S4-S6.

**Figure 2:**
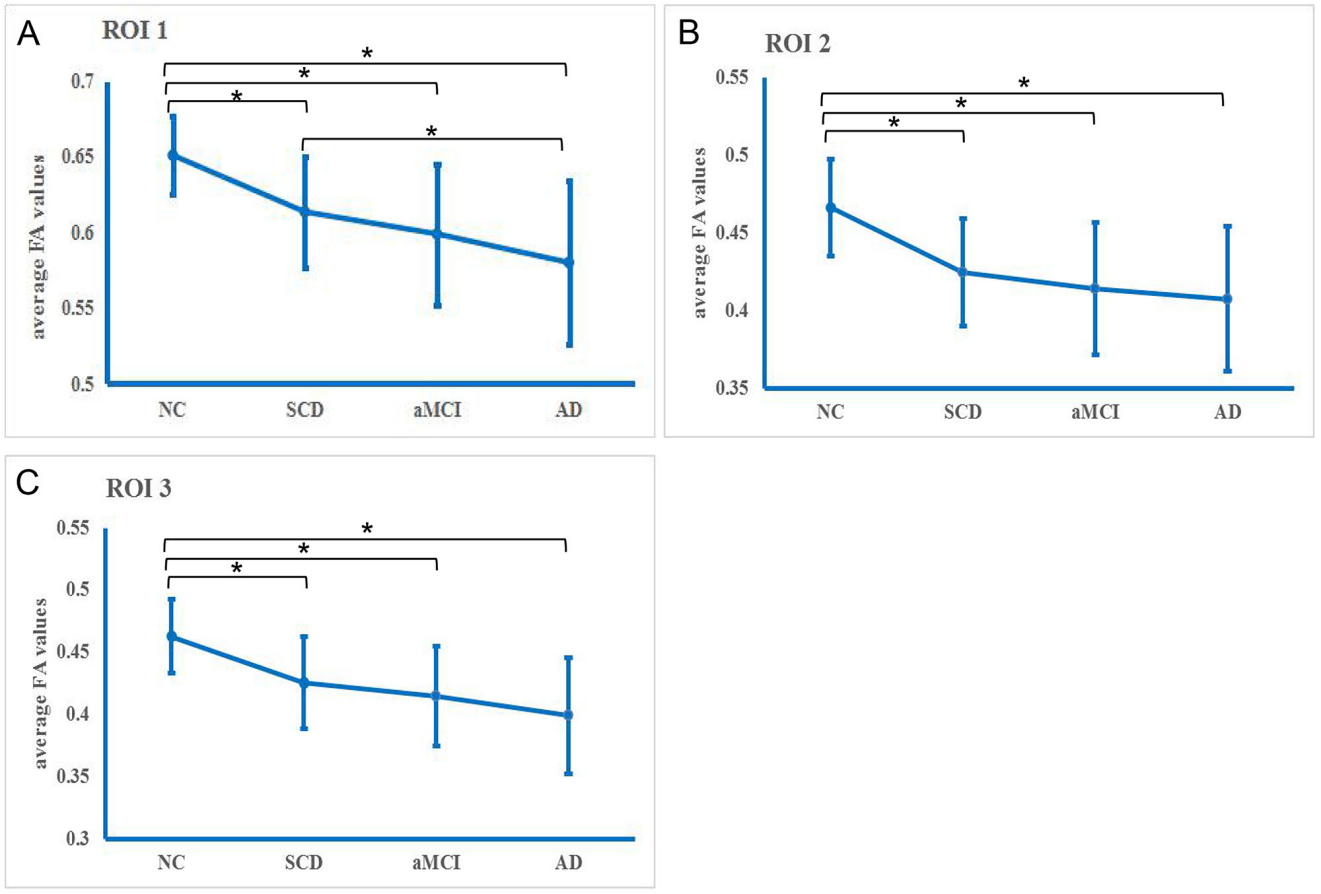
Averaged fractional anisotropy (FA) in the common white matter impairment regions in each diagnostic group **A.** Region of interest (ROI) 1, **B.** ROI 2, and **C.** ROI 3. * Significance (p<0.05).

The common WM impairments tracts in the three patient groups were denoted as region of interest (ROI) 1, ROI 2 and ROI 3 (Table 2). For every ROI, the averaged FA values of all the subjects in each group are presented in Figure 2 & Supplementary Table S7. Differences in the averaged FA values for every ROI were observed among the four groups (all p < 0.05). Post hoc comparisons revealed decreased averaged FA values in the three patient groups relative to the NC group. In ROI 1, there was also an increased averaged FA value in the SCD group compared with the AD group.

**Table 2:** Common white matter impairment ROIs

	Cluster size	Tracts contained in this cluster
ROI 1	4148	body, genu and splenium of CC, bilateral ACR and SCR, PCR L
ROI 2	715	ACR R, SLF R
ROI 3	602	ACR L, SLF L

CC, corpus callosum; ACR, Anterior corona radiata; SCR, Superior corona radiata; PCR, Posterior corona radiata; SLF, Superior longitudinal fasciculus; L, left; R, right.

### Relationship between DTI and neuropsychological testing

**Figure 3:**
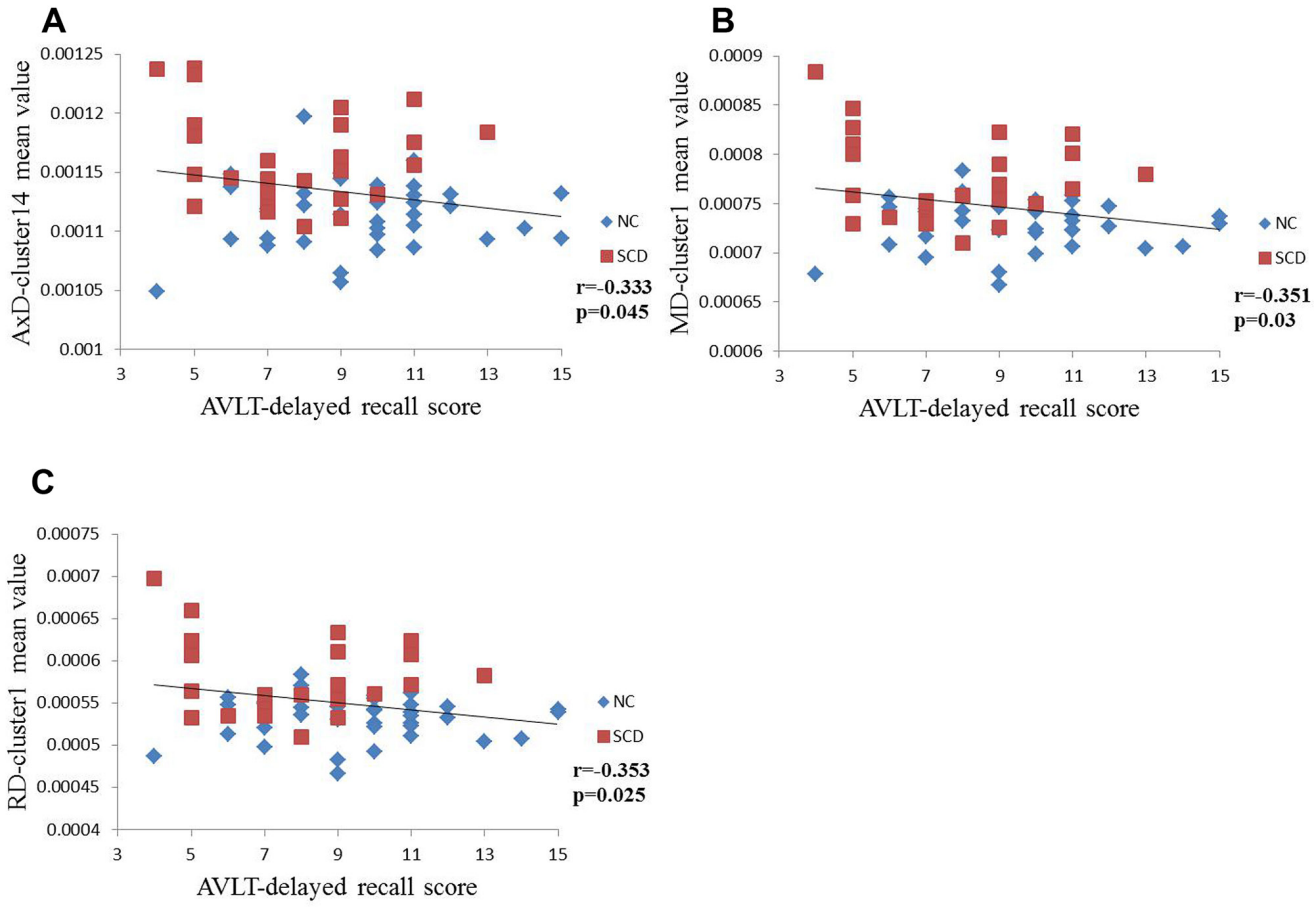
Scatterplots illustrating the relationship between AVLT-delayed recall score for NC and SCD patients and, A. AxD, B. MD, or C. RD Significance, p<0.05 (after Bonferroni correction for the number of cognitive test variables).

In each extracted cluster, correlations between DTI parameters and neuropsychological test scores were investigated. After Bonferroni correction, the AVLT-delayed recall score was correlated with higher AxD (r=−0.333, p=0.045), MD (r=−0.351, p=0.03) and RD (r=−0.353, p=0.025) in the NC and SCD groups (Figure 3). However, in the aMCI and AD groups, DTI parameters were strongly correlated with neuropsychological test scores. Thus, lower FA and higher diffusivity parameters (MD, RD and AxD) were correlated with worse cognitive performance (Figure 3, Table 3).

**Table 3:** Partial correlation coefficients for DTI parameters and neuropsychological test results (controlled for age, gender and years of education)

		MMSE	MoCA	AVLT- immediate recall	AVLT-delayed recall	AVLT-recognition
SCD	FA-cluster 1	0.013	0.178	0.107	0.256	0.197
	AxD-cluster 14	−0.274	−0.216	−0.132	−0.333*	−0.139
	AxD-cluster 13	−0.125	−0.18	−0.112	−0.287	−0.072
	AxD-cluster 12	−0.174	−0.304	−0.088	−0.265	−0.053
	AxD-cluster 11	0.131	−0.025	−0.042	−0.13	0.111
	AxD-cluster 10	−0.017	−0.028	0.027	0.025	0.068
	MD-cluster 1	−0.158	−0.212	−0.105	−0.351*	−0.122
	RD-cluster 1	−0.115	−0.208	−0.099	−0.353*	−0.149
aMCI	FA-cluster 1	0.220	0.462*	0.462*	0.574*	0.338*
	AxD-cluster 1	−0.159	−0.362*	−0.351*	−0.420*	−0.178
	MD-cluster 1	−0.201	−0.420*	−0.429*	−0.533*	−0.289*
	RD-cluster 2	−0.247	−0.452*	−0.461*	−0.589*	−0.353*
AD	FA-cluster 1	0.721*	0.675*	0.687*	0.686*	0.717*
	AxD-cluster 2	−0.774*	−0.758*	−0.750*	−0.741*	−0.716*
	MD-cluster 1	−0.807*	−0.770*	−0.751*	−0.74*	−0.754*
	RD-cluster 2	−0.803*	−0.765*	−0.742*	−0.736*	−0.757*

* Indicates significance (p<0.05 after Bonferroni correction for the number of cognitive test variables). Tracts that were included in each cluster were shown in Supplementary Tables S1-S3. aMCI, Amnestic Mild Cognitive Impairment; AD, Alzheimer's disease; MMSE, Mini-Mental State Examination; MoCA, Montreal Cognitive Assessment; AVLT, Auditory Verbal Learning Test; FA, fractional anisotropy; MD, mean diffusivity; AxD, axial diffusivity; RD, radial diffusivity.

## DISCUSSION

In the present study, we investigated the WM characteristics of SCD patients and found that the SCD group had detectable microstructural alterations in WM, intermediate to the NC group and the other two patient groups. Several of the impaired WM tracts connect structures involved in the early stages of AD, such as the hippocampus, the medial temporal lobe, and the posterior cingulate cortex [12, 31, 32]. Our results were generally consistent with previous research [19, 33], whose results suggested that underlying neurodegenerative changes have already occurred at the SCD stage and that DTI can contribute additional information at this stage. Kiuchi and colleagues found no differences between NC and SCD subjects in DTI measures and suggested that their results were in agreement with the hypothetical model of the dynamic biomarkers of AD [23]. However, the SCD group in their study had equal or better memory performance when compared with NC. Considering the unspecific nature of SCD, previous studies may have included a large proportion of SCD due to other conditions instead of AD. In the present study, meeting the research criteria for pre-MCI SCD proposed by SCD-I gave us more confidence in our results than previously published results.

We found that SCD patients had decreased FA and increased MD, AxD, and RD when compared with NC, and tracts showing differences in these DTI parameters were overlapping. Since increased RD may be associated with myelin damage [27–30], our results suggest that the demyelination might be mainly responsible for the WM integrity decline in SCD, though further validation is still needed. Axonal injury might also play a minor role in the SCD group, according to the increased AxD in localized WM tracts.

We observed an increased averaged FA value in the SCD group compared with the AD group in ROI 1; moreover, there was an averaged FA downward trend consistent with the hypothesized disease continuum of SCD-aMCI-AD in the other common WM impairment ROIs. Generally consistent with previous findings [19, 34], our results suggest that the destruction of WM integrity might have already occurred at the SCD stage and progresses further at the aMCI and AD stages. Thus, FA may be useful for monitoring AD progression [35, 36].

We found correlations between DTI findings and AVLT delayed recall scores when comparing the NC and SCD groups. Across the NC and aMCI or AD groups, DTI findings were associated with scores of AVLT immediate recall, delayed recall, and recognition. AVLT is one of the most sensitive and widely used episodic memory tests [37]. DTI findings had strong associations with AVLT scores, suggesting that WM microstructure alterations may contribute to memory impairment across the patient groups. Our correlation results across the NC and SCD groups support the idea that WM impairment is the strongest structural predictor of delayed recall score, especially in the early stages of AD [38]. Previous studies suggested that the delayed recall test best discriminates early AD and predicts conversion to AD [39, 40]. Thus, the decreasing trend of delayed recall in SCD patients implied the presence of preclinical AD in the SCD group. DTI parameters were not correlated with the scores of MMSE and MoCA across the NC and SCD groups. Considering the fact that SCD patients have partially successful compensation, this may make it difficult for those standardized cognitive test to differentiate it from normal performance [1] and may account for this finding.

There are some limitations of our study should be taken into account. First, our sample size was small. Future analyses of more participants should be performed to confirm the present results. Second, our study was cross-sectional; further longitudinal studies are therefore needed to define changes in DTI metrics and cognitive function with disease evolution. Third, we did not combine gray matter changes and cerebrospinal fluid biomarkers in our study, which together could provide a more comprehensive understanding of the neuropathological mechanism of early AD. Nonetheless, our results suggest that WM abnormalities can be detected by DTI measures in the SCD group, and they are less severe than the aMCI and AD groups. These WM alterations may contribute to the early memory decline perceived by SCD subjects and subsequent cognitive dysfunction.

## MATERIALS AND METHODS

### Participants

124 right-handed, Han Chinese subjects were enrolled in this study. Twenty-seven SCD patients, thirty-five aMCI patients, and twenty-five AD patients were recruited from the memory clinic of the Neurology Department, Xuanwu Hospital, Capital Medical University, Beijing, China. Thirty-seven NC subjects were recruited from the local community by advertisements. All subjects provided written informed consent before enrollment. All of the subjects underwent a series of standardized clinical evaluations (see Table 1), which included the Chinese version of MMSE [41], MoCA the Beijing version [42], Clinical Dementia Rating Scale (CDR) [43] and the World Health Organization-University of California Los Angeles AVLT [44].

All SCD patients met the research criteria for pre-MCI SCD proposed by SCD-I[1]: (a) presence of self-perceived continuous cognitive decline compared to previous normal status within the last 5 years combined with informant report; (b) normal performance on both MMSE and MoCA after age-, gender-, and education-adjustment; and (c) CDR score of 0.

The aMCI subjects met the criteria proposed in 2001 [45]: (a) memory complaint, preferably confirmed by an informant; (b) objective memory impairment; (c) normal or near-normal performance on general cognitive functioning and no or minimum impairment of daily life activities; (d) CDR score of 0.5; and (e) failure to meet the criteria for dementia according to the DSM-IV.

The diagnosis of AD fulfilled published diagnostic criteria [46–48]: (a) meeting for dementia; (b) gradual and progressive change in memory function over more than 6 months; (c) impaired episodic memory on objective testing; and (d) hippocampal atrophy confirmed by structural MRI.

Criteria for NC were defined as: (a) having no report of any cognition complaint; and (b) normal performance on both MMSE and MoCA after age-, gender-, and education-adjusted and CDR scored 0.

Exclusion criteria for all the subjects were: (a) a history of stroke (Hachinski Ischemic Scale > 4); (b) severe depression (Hamilton Depression Rating Scale score > 24 or the centre for Epidemiological Studies Depression Scale > 21); (c) other central nervous system diseases which could cause cognitive decline (e.g., brain tumors, Parkinson's disease, encephalitis, or epilepsy); (d) other diseases which could cause cognitive decline (e.g., thyroid dysfunction, severe anemia, syphilis, or HIV); (e) a history of psychosis or congenital mental growth retardation; (f) cognitive decline caused by traumatic brain injury; or (g) those who could not complete neuropsychological tests or with contraindication to MRI.

### DTI acquisition

All MRI examinations were performed using a 3.0-Tesla Magnetom Trio Tim scanner (Siemens, Erlangen, Germany). The session started with the acquisition of a T1-weighted three-dimensional magnetization-prepared rapid gradient echo (MP-RAGE). The parameters were: repetition time/echo time/inversion time (TR/TE/TI) = 1900ms/2.2ms/900ms, flip angle=9°, matrix=256× 256, field of view (FOV) = 256 × 256 mm^2^, sagittal slices = 176, thickness = 1 mm, and voxel size = 1 × 1 × 1 mm^3^. The single shot spin echo-echo planar imaging (SS-SE-EPI) sequence was used in the DTI scans. Diffusion weighted images (DWI) were acquired along 30 non-collinear and non-coplanar directions with b=1000 s/mm^2^ and one b=0 s/mm^2^ image. The parameters were: flip angle=90°, FOV=256×256mm^2^, matrix=128×128, 60 slices with 2 mm slice thickness.

### DTI imaging analysis

The Oxford Centre for Functional MRI of the Brain (FMRIB) Software Library (FSL 5.0, http://fsl.fmrib.ox.ac.uk/fsl/fslwiki/) [49–51] was used for raw DTI data analyses and calculations. First, for data pre-processing: (1) the EddyCorrect tool was used for correcting head motion and eddy current distortions by fine registration of the DTI images of the low-b (b value = 0 s/mm^2^) image; (2) brain masks of all the subjects were created using the Brain Extraction Tool (BET) [49]; and (3) using the least-squares algorithm fitting tensor model included in the DTI-FIT Tool [52], a diffusion tensor, or ellipsoid, was modeled at each voxel. Based on the eigenvalues of the tensor, FA, MD, AD, and RD values were calculated on a voxel by voxel basis.

Voxel-wise analysis of the DTI parameters (FA, MD, AxD, and RD) was performed using Tract-Based Spatial Statistics (TBSS) [53]. Using FSL's nonlinear image registration algorithm, all subjects' FA maps were aligned into a 1×1×1 mm^3^ standard Montreal Neurological Institute (MNI) 152 space. The target template was the FMRIB58_FA (http://www.fmrib.ox.ac.uk/fsl/data/FMRIB58_FA). Then a mean FA image was created by averaging the aligned FA maps. The mean FA image was thinned to create a mean FA skeleton representing the center of all tracts common to all participants in the present study. Each subject's aligned FA data were later projected onto the FA skeleton to obtain their FA skeletons and deformation matrixes. With the deformation matrixes, the skeletonized AxD, MD, and RD maps were created by the tbss_non_FA tool. The skeletonized FA, AxD, MD, and RD map images were subsequently fed to statistical analysis. ICBM-DTI-81 parcellation map [54] was applied to identify the names of WM tracts that contained the clusters of significant between-group differences. Index of ROIs from the ICBM-DTI-81 white-matter labels atlas followed by their abbreviations are present in Supplementary Table S8.

### Statistics

To uncover WM impairments in the SCD, aMCI and AD patients, three group comparisons (SCD vs. NC, aMCI vs. NC and AD vs. NC) were performed on the skeletonized DTI maps. Group differences were detected by permutation tests with the Randomise tool in FSL (http://fsl.fmrib.ox.ac.uk/fsl/fslwiki/Randomise). The number of permutations was set to 5000. Correction for multiple comparisons was estimated using family wise error (FWE) and the threshold-free cluster enhancement (TFCE) option. Unless otherwise noted, results are reported at P<0.05 (TFCE and FWE corrected, voxels >100). For better visualization, the TBSS results were thickened with the tbss_fill tool provided by FSL. The WM impairments of the patient groups were represented by the significant FA alterations. Based on the WM impairments of the SCD, aMCI, and AD patients, we obtained the common WM impairments tracts from the three groups. The common WM impairments regions containing at least 500 voxels were considered as ROIs. The mean FA values of all the voxels in each ROI were calculated for all subjects. To control for false positive results, the common WM impairments regions were required to pass significance of P<0.005 (TFCE and FWE corrected, voxels >500). For the subjects in NC, SCD, aMCI, and AD, the mean FA values were then averaged in each group to demonstrate alterations of FA values across the four groups. For group effects in those averaged FA values, comparisons were performed among four groups using one-way ANOVA with post hoc tests and Bonferroni correction.

To investigate the relationship between the WM impairment and cognitive performance, we performed two-sample t test between the patients and normal controls on FA, AxD, MD and RD values. The results surviving the threshold (TFCE and FWE corrected, voxels>100) were named as “clusters”. For FA, AxD, MD and RD values, we calculate the mean values of all the voxels in each cluster. Then, we carried out correlation analysis between the mean DTI values and neuropsychological test scores controlling for age, gender and education between each patient group and the NC group. The Partial correlation of the Statistical Package for Social Science (SPSS, v. 18.0) (http://www-01.ibm.com/software/analytics/spss/) was employed for correlation analysis. Significance was set at P<0.05. The p values were Bonferroni-corrected for the number of cognitive tests (MMSE, MoCA, AVLT-immediate recall, delayed recall and recognition) investigated.

## SUPPLEMENTARY FIGURES AND TABLES


