# Supplementary material for: White matter degeneration in subjective cognitive decline: a diffusion tensor imaging study
Source: Oncotarget. 2016 Jun 15;7(34):54405–14. doi: 10.18632/oncotarget.10091 (PMC5342351; doi:10.18632/oncotarget.10091)
Supplement: Supplementary file 1 [file oncotarget-07-54405-s001.pdf]

White matter degeneration in subjective cognitive decline: a diffusion tensor imaging study

SUPPLEMENTARY FIGURES AND TABLES

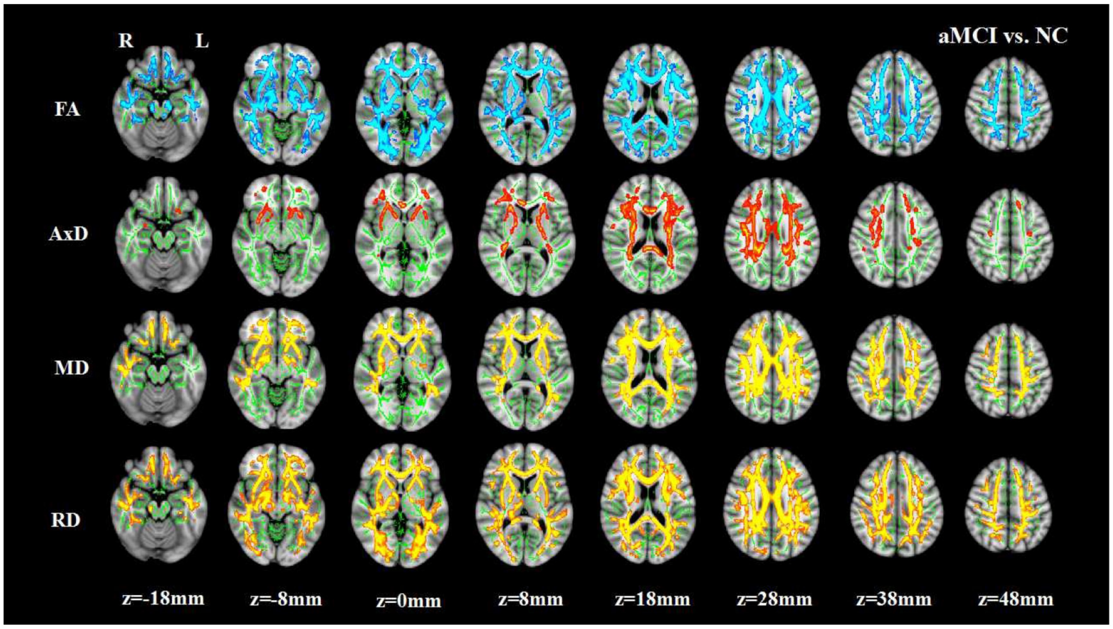

Supplementary Figure S1: Group differences between normal controls (NC) and amnestic mild cognitive impairment (aMCI). The brain images showing underlying standard Montreal Neurological Institute (MNI) atlas MNI152 1-mm brain template and white matter skeleton derived from tract-based spatial statistics (TBSS) analysis (shown in green).Blue-Light Blue color indicates tracts with decreased fractional anisotropy (FA), Red-Yellow color indicates tracts with increased axial diffusivity (AxD), mean diffusivity (MD) and radial diffusivity (RD) in aMCI vs. NC, respectively. The threshold for results was set at $P<0.05$ (TFCE and FWE corrected, voxels>100).

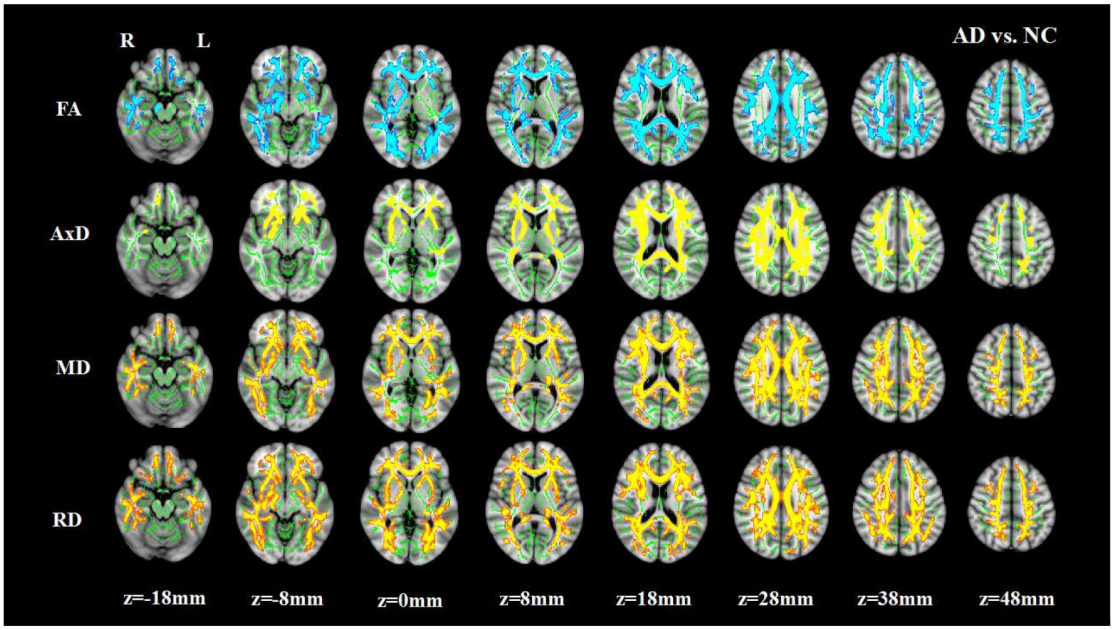

Supplementary Figure S2: Group differences between normal controls (NC) and Alzheimer's disease (AD). The brain images showing underlying standard Montreal Neurological Institute (MNI) atlas MNI152 1-mm brain template and white matter skeleton derived from tract-based spatial statistics (TBSS) analysis (shown in green).Blue-Light Blue color indicates tracts with decreased fractional anisotropy (FA), Red-Yellow color indicates tracts with increased axial diffusivity (AxD), mean diffusivity (MD) and radial diffusivity (RD) in AD vs. NC, respectively. The threshold for results was set at $P<0.05$ (TFCE and FWE corrected, voxels>100).

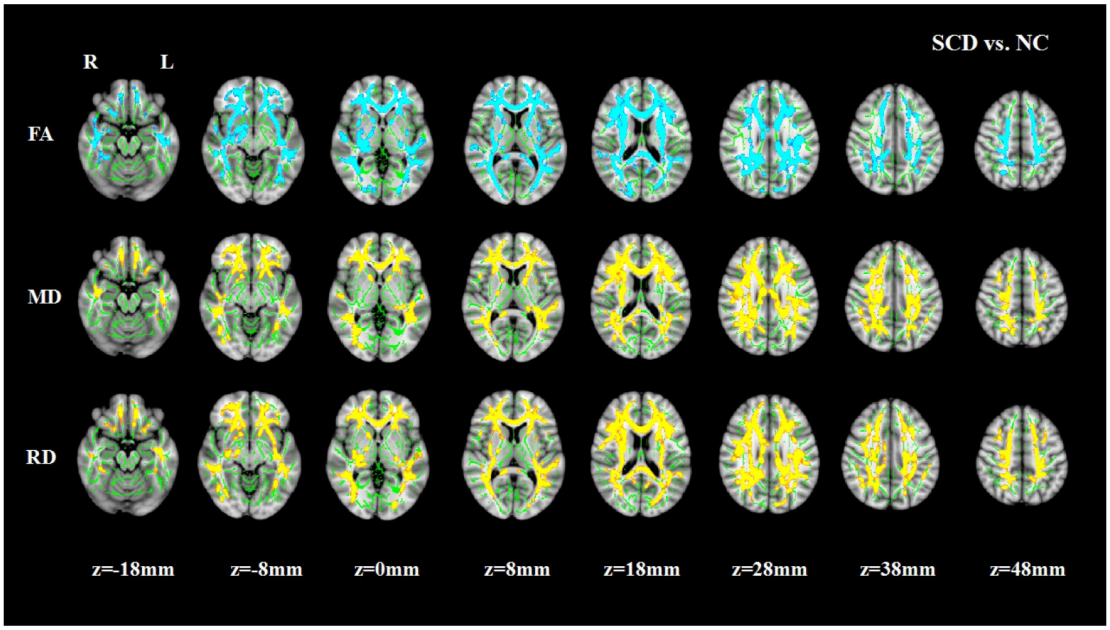

Supplementary Figure S3: Group differences between normal controls (NC) and subjective cognitive decline (SCD). The brain images showing underlying standard Montreal Neurological Institute (MNI) atlas MNI152 1-mm brain template and white matter skeleton derived from tract-based spatial statistics (TBSS) analysis (shown in green). Blue-Light blue color indicates tracts with decreased fractional anisotropy (FA), Red-Yellow color indicates tracts with increased mean diffusivity (MD) and radial diffusivity (RD) in SCD vs. NC, respectively. For axial diffusivity (AxD), no voxels were significantly different between SCD and NC. The threshold for results was set at $P<0.005$ (TFCE and FWE corrected, voxels>500).

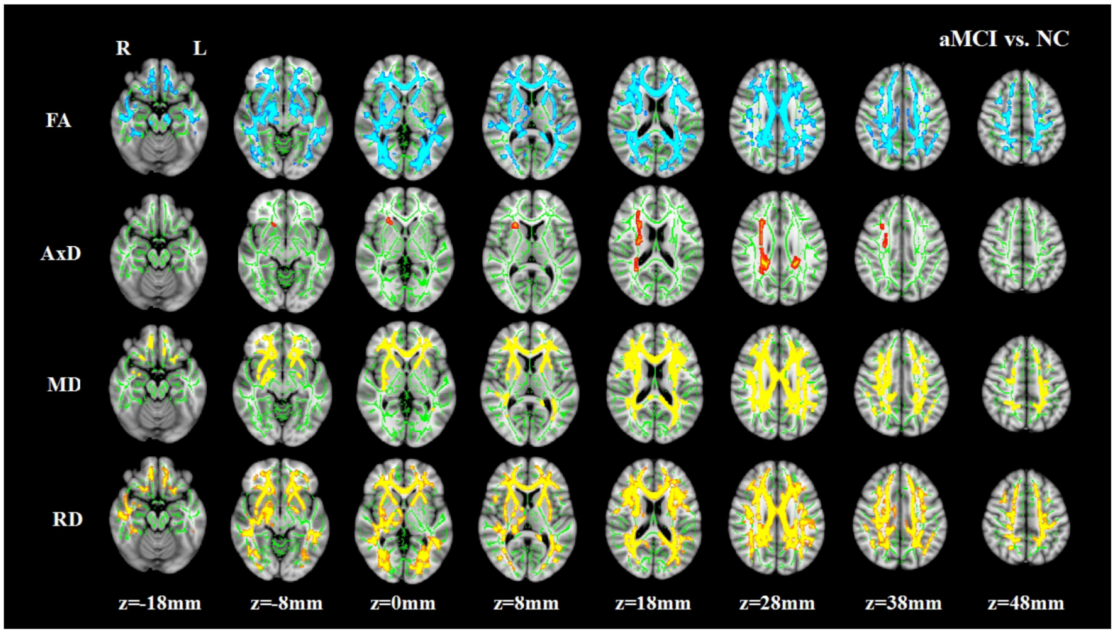

Supplementary Figure S4: Group differences between normal controls (NC) and amnestic mild cognitive impairment (aMCI). The brain images showing underlying standard Montreal Neurological Institute (MNI) atlas MNI152 1-mm brain template and white matter skeleton derived from tract-based spatial statistics (TBSS) analysis (shown in green).Blue-Light Blue color indicates tracts with decreased fractional anisotropy (FA), Red-Yellow color indicates tracts with increased axial diffusivity (AxD), mean diffusivity (MD) and radial diffusivity (RD) in aMCI vs. NC, respectively. The threshold for results was set at P<0.005 (TFCE and FWE corrected, voxels>500).

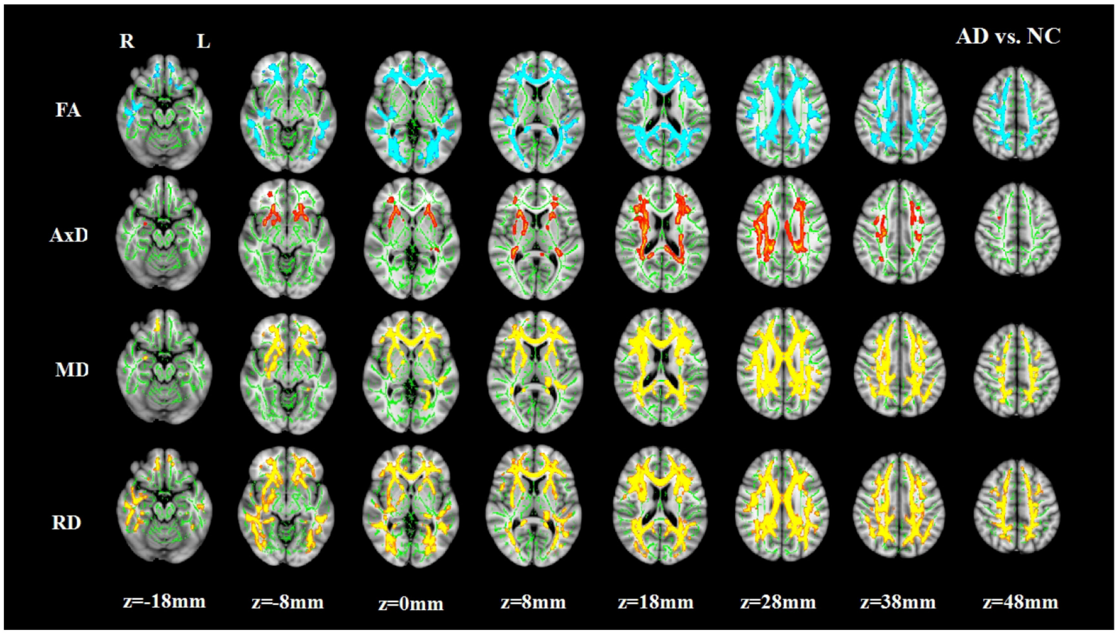

Supplementary Figure S5: Group differences between normal controls (NC) and Alzheimer's disease (AD). The brain images showing underlying standard Montreal Neurological Institute (MNI) atlas MNI152 1-mm brain template and white matter skeleton derived from tract-based spatial statistics (TBSS) analysis (shown in green). Blue-Light Blue color indicates tracts with decreased fractional anisotropy (FA), Red-Yellow color indicates tracts with increased axial diffusivity (AxD), mean diffusivity (MD) and radial diffusivity (RD) in AD vs. NC, respectively. The threshold for results was set at P<0.005 (TFCE and FWE corrected, voxels>500).

Supplementary Table S1: Results of two sample t test between SCD and normal controls

Tracts	FA-cluster1	AxD-cluster14	AxD -cluster13	AxD -cluster12	AxD -cluster11	AxD -cluster10	MD-cluster1	RD-cluster1
CC	g,b,s	g,b,s	g				g,b,s	g,b,s
cbFN								
CT	Bilateral							
CP	Bilateral						R	R
ALIC	Bilateral	R					Bilateral	Bilateral
PLIC	Bilateral	R					Bilateral	Bilateral
RIC	Bilateral	R			L		Bilateral	Bilateral
ACR	Bilateral	R	L				Bilateral	Bilateral
SCR	Bilateral	R					Bilateral	Bilateral
PCR	Bilateral	R					Bilateral	Bilateral
PTR	Bilateral	R			L		Bilateral	Bilateral
SS	Bilateral	R		R			Bilateral	Bilateral
EC	Bilateral	R		R			Bilateral	Bilateral
CCG	Bilateral	R					R	Bilateral
CH								
F/ST	Bilateral						Bilateral	Bilateral
SLF	Bilateral	R	L		L		Bilateral	Bilateral
SFOF	Bilateral						Bilateral	Bilateral
UF	Bilateral						Bilateral	Bilateral
TAP	R	R					Bilateral	Bilateral
ML	Bilateral							
MLF								
ICP								
MCP	√							
SCP	Bilateral							
IFO/UNC								
IFO/ILF								
AC								

The threshold for results was set at P<0.05 (TFCE and FWE corrected, voxels>100).
SCD, subjective cognitive decline; FA, fractional anisotropy; MD, mean diffusivity; RD, radial diffusivity. R, right; L, left.

Supplementary Table S2: Results of two sample t test between aMCI and normal controls

Tracts	FA-cluster1	AxD-cluster1	MD-cluster1	RD-cluster2
CC	g,b,s	g,b,s	g,b,s	g,b,s
cbFN				
CT	Bilateral			R
CP	Bilateral		L	Bilateral
ALIC	Bilateral	Bilateral	Bilateral	Bilateral
PLIC	Bilateral	Bilateral	Bilateral	Bilateral
RIC	Bilateral	Bilateral	Bilateral	Bilateral
ACR	Bilateral	Bilateral	Bilateral	Bilateral
SCR	Bilateral	Bilateral	Bilateral	Bilateral
PCR	Bilateral	Bilateral	Bilateral	Bilateral
PTR	Bilateral	Bilateral	Bilateral	Bilateral
SS	Bilateral		Bilateral	Bilateral
EC	Bilateral	Bilateral	Bilateral	Bilateral
CCG	Bilateral		Bilateral	Bilateral
CH				L
F/ST	Bilateral		R	Bilateral
SLF	Bilateral	Bilateral	Bilateral	Bilateral
SFOF	Bilateral	Bilateral	Bilateral	Bilateral
UF	Bilateral		Bilateral	Bilateral
TAP	Bilateral	Bilateral	Bilateral	Bilateral
ML				
MLF				
ICP				
MCP	√			√
SCP	L			
IFO/UNC				
IFO/ILF				
AC				

The threshold for results was set at $P<0.05$ (TFCE and FWE corrected, voxels>100).
aMCI, amnestic mild cognitive impairment; FA, fractional anisotropy; AxD, axial diffusivity; MD, mean diffusivity; RD, radial diffusivity. R, right; L, left.
"√" indicates this region is activated but can not be divided into right and left part.

Supplementary Table S3: Results of two sample t test between AD and normal controls

Tracts	FA-cluster1	AxD-cluster2	MD-cluster1	RD-cluster2
CC	g,b,s	g,b,s	g,b,s	g,b,s
cbFN				
CT	R			
CP	R			R
ALIC	R	Bilateral	Bilateral	Bilateral
PLIC	R	Bilateral	Bilateral	Bilateral
RIC	Bilateral	Bilateral	Bilateral	Bilateral
ACR	Bilateral	Bilateral	Bilateral	Bilateral
SCR	Bilateral	Bilateral	Bilateral	Bilateral
PCR	Bilateral	Bilateral	Bilateral	Bilateral
PTR	Bilateral	Bilateral	Bilateral	Bilateral
SS	Bilateral		Bilateral	Bilateral
EC	Bilateral	Bilateral	Bilateral	Bilateral
CCG	Bilateral	R	Bilateral	Bilateral
CH			L	Bilateral
F/ST	R	L	Bilateral	Bilateral
SLF	Bilateral	Bilateral	Bilateral	Bilateral
SFOF		Bilateral	Bilateral	Bilateral
UF	L	R	Bilateral	Bilateral
TAP	Bilateral	Bilateral	Bilateral	Bilateral
ML				
MLF				
ICP				
MCP	√			
SCP				
IFO/UNC				
IFO/ILF				
AC				

The threshold for results was set at P<0.05 (TFCE and FWE corrected, voxels>100).
AD, Alzheimer's disease; FA, fractional anisotropy; AxD, axial diffusivity; MD, mean diffusivity; RD, radial diffusivity.
R, right; L, left.

Supplementary Table S4: Results of two sample t test between SCD and normal controls

Tracts	FA-cluster1	MD-cluster4	MD-cluster3	MD-cluster2	RD-cluster7
CC	g, b, s	g,b,s	b,s		g, b, s
cbFN					
CT					
CP	R				R
ALIC	Bilateral	Bilateral			Bilateral
PLIC	Bilateral	Bilateral			Bilateral
RIC	Bilateral	R	L		Bilateral
ACR	Bilateral	Bilateral			Bilateral
SCR	Bilateral	Bilateral	L		Bilateral
PCR	Bilateral	R	L		Bilateral
PTR	Bilateral	R	L		Bilateral
SS	Bilateral	R	L	R	Bilateral
EC	Bilateral	Bilateral		R	Bilateral
CCG	R	R			R
CH					
F/ST	Bilateral		L		Bilateral
SLF	Bilateral	Bilateral	L		Bilateral
SFOF	Bilateral	Bilateral			Bilateral
UF	L				L
TAP	R	R			R
ML					
MLF					
ICP					
MCP					
SCP					
IFO/UNC					
IFO/ILF					
AC					

The threshold for results was set at P<0.005 (TFCE and FWE corrected, voxels>500).
SCD, subjective cognitive decline; FA, fractional anisotropy; MD, mean diffusivity; RD, radial diffusivity. R, right; L, left.

Supplementary Table S5: Results of two sample t test between aMCI and normal controls

Tracts	FA-cluster1	AxD-cluster4	AxD-cluster3	MD-cluster4	RD-cluster1
CC	g, b, s		b, s	g, b, s	g, b, s
cbFN					
CT	Bilateral				
CP	Bilateral				R
ALIC	Bilateral	R		Bilateral	Bilateral
PLIC	Bilateral	R		Bilateral	Bilateral
RIC	Bilateral		R	Bilateral	Bilateral
ACR	Bilateral	R		Bilateral	Bilateral
SCR	Bilateral	R	R	Bilateral	Bilateral
PCR	Bilateral	R		Bilateral	Bilateral
PTR	Bilateral			Bilateral	Bilateral
SS	Bilateral				Bilateral
EC	Bilateral	R		Bilateral	Bilateral
CCG	Bilateral			Bilateral	Bilateral
CH					
F/ST	Bilateral			R	R
SLF	Bilateral			Bilateral	Bilateral
SFOF	Bilateral			Bilateral	Bilateral
UF	Bilateral			Bilateral	Bilateral
TAP	Bilateral		R	Bilateral	Bilateral
ML					
MLF					
ICP					
MCP	√				
SCP					
IFO/UNC					
IFO/ILF					
AC					

The threshold for results was set at P<0.005 (TFCE and FWE corrected, voxels>500).
aMCI, amnestic mild cognitive impairment; FA, fractional anisotropy; AxD, axial diffusivity; MD, mean diffusivity; RD, radial diffusivity. R, right; L, left.
"√" indicates this region is activated but can not be divided into right and left part.

Supplementary Table S6: Results of two sample t test between AD and normal controls

Tracts	FA-cluster1	AxD-cluster2	AxD-cluster1	MD-cluster1	RD-cluster1
CC	g, b, s		g, b, s	g, b, s	g, b, s
cbFN					
CT					
CP					
ALIC		R	L	Bilateral	Bilateral
PLIC		R	L	Bilateral	Bilateral
RIC	Bilateral	R	L	Bilateral	Bilateral
ACR	Bilateral	R	L	Bilateral	Bilateral
SCR	Bilateral	R	L	Bilateral	Bilateral
PCR	Bilateral	R	L	Bilateral	Bilateral
PTR	Bilateral	R	L	Bilateral	Bilateral
SS	Bilateral				Bilateral
EC	Bilateral	R	L	Bilateral	Bilateral
CCG	Bilateral			Bilateral	Bilateral
CH				L	Bilateral
F/ST	R		L	Bilateral	Bilateral
SLF	Bilateral	R	L	Bilateral	Bilateral
SFOF		R	L	Bilateral	Bilateral
UF		R		R	R
TAP	Bilateral	R	L	Bilateral	Bilateral
ML					
MLF					
ICP					
MCP					
SCP					
IFO/UNC					
IFO/ILF					
AC					

The threshold for results was set at P<0.005 (TFCE and FWE corrected, voxels>500).
AD, Alzheimer's disease; FA, fractional anisotropy; AxD, axial diffusivity; MD, mean diffusivity; RD, radial diffusivity.
R, right; L, left.

Supplementary Table S7: Fractional anisotropy (FA) values in those common impairment ROIs

	NC	SCD	aMCI	AD
ROI 1	0.651±0.026	0.614±0.037	0.599±0.047	0.580±0.054
ROI 2	0.466±0.031	0.424±0.035	0.414±0.043	0.408±0.047
ROI 3	0.463±0.030	0.425±0.037	0.415±0.040	0.399±0.047

Data are presented as the mean ± SD. NC, normal control; SCD, Subjective cognitive decline; aMCI, Amnestic Mild Cognitive Impairment; AD, Alzheimer’s disease.

Supplementary Table S8: Index of ROIs from the ICBM-DTI-81 white-matter labels atlas followed by their abbreviations

Genu of corpus callosum	gCC	Cingulum (cingulate gyrus)	CCG L,R
Body of corpus callosum	bCC	Cingulum (hippocampus)	CH L,R
Splenium of corpus callosum	sCC	Fornix (cres)/Stria terminalis	F/ST L,R
Fornix (column and body of fornix)	cbFN	Superior longitudinal fasciculus	SLF L,R
Corticospinal tract	CT L,R	Superior fronto-occipital fasciculus	SFOF L,R
Cerebral peduncle	CP L,R	Uncinate fasciculus	UF L,R
Anterior limb of internal capsule	ALIC L,R	Tapetum	TAP L,R
Posterior limb of internal capsule	PLIC L,R	Medial lemniscus	ML
Retrolenticular part of internal capsule	RIC L,R	Medial longitudinal fasciculus	MLF
Anterior corona radiata	ACR L,R	Inferior cerebellar peduncle	ICP
Superior corona radiata	SCR L,R	Middle cerebellar peduncle	MCP
Posterior corona radiata	PCR L,R	Superior cerebellar peduncle	SCP
Posterior thalamic radiation	PTR L,R	Inferior fronto-occipital fasciculus/ Uncinate fasciculus	IFO/ UNC
Sagittal stratum	SS L,R	Inferior fronto-occipital fasciculus/ Inferior longitudinal fasciculus	IFO/ ILF
External capsule	EC L,R	Anterior commissure	AC
